# *Borrelia valaisiana* – a candidate human pathogen? Insights from the tick-borne diseases STING study

**DOI:** 10.1186/s12879-026-13620-z

**Published:** 2026-05-22

**Authors:** Julia Levin, Malin Lager, Marika Nordberg, Dag Nyman, Pia Forsberg, Per-Eric Lindgren, Anna J Henningsson, Peter Wilhelmsson, Johanna Sjöwall

**Affiliations:** 1 Clinical Department of Infectious Diseases in Östergötland, Region Östergötland, Norrköping, Sweden; 2 Clinical Department of Infectious Diseases, Region Jönköping County, Jönköping, Sweden; 3 National Reference Laboratory for Borrelia and Other Tick-Borne Bacteria, Division of Clinical Microbiology, Laboratory Medicine, Region Jönköping County, Jönköping, Sweden; 4 Åland Health Services, Mariehamn, Åland Finland; 5 The Borrelia Research Group of the Åland Islands, Mariehamn, Åland Finland; 6 Department of Biomedical and Clinical Sciences, Linköping University, Linköping, Sweden

**Keywords:** *Borrelia valaisiana*, Human, Ticks, Seroconversion, Symptoms, Infection

## Abstract

**Background:**

*Borrelia* (*B*.) *valaisiana* is a tick-borne spirochete within the *Borrelia burgdorferi* sensu lato complex. In Sweden, it occurs in *Ixodes ricinus* ticks, but its role as a human pathogen remains uncertain. In the Tick-Borne Diseases STING study conducted during 2007–2015, adult participants from Sweden and the Åland Islands, Finland, provided ticks, blood samples for serological analyses, and questionnaires at inclusion and at a 3-month follow-up. Medical records were reviewed for those seeking care. Ticks were characterised, feeding duration assessed, and pathogens detected by real-time PCR. During 2008–2009, *B. valaisiana* was identified by PCR in 1.6% (34/2,154) of the ticks. Since its significance as a human pathogen is unclear, we chose to examine this tick-bitten cohort in greater detail. Accordingly, the objective of the study was to assess whether participants bitten by *B. valaisiana*-positive ticks developed Borrelia-specific antibodies and/or symptoms suggestive of infection.

**Materials and methods:**

Participants bitten by *B. valaisiana*-positive ticks (VPOS, *n* = 33; one bitten twice) were compared with an age- and sex-matched group bitten by Borrelia-negative ticks (VNEG, *n* = 67). Borrelia-specific IgG was analysed in paired blood samples.

**Results:**

Median age in VPOS was 67 years; 58% were women. Seroconversion for Borrelia-specific IgG occurred significantly more often in VPOS than VNEG (*p* = 0.03). Common symptoms in VPOS included neck pain, myalgia/arthralgia, numbness, and headache, but none sought medical care. No significant difference in symptom occurrence was observed between groups.

**Conclusion:**

Tick bites involving *B. valaisiana* were associated with higher Borrelia antibody seroconversion, without significant symptom associations or need for medical intervention. The results of this study provide new insights into the potential human impact of *B. valaisiana*.

**Supplementary Information:**

The online version contains supplementary material available at 10.1186/s12879-026-13620-z.

## Introduction

*Borrelia* (*B*.) *valaisiana* is a tick-borne spirochete belonging to the *Borrelia burgdorferi* sensu lato (s.l.) complex [1, 2]. It is the third most common *Borrelia* species found in ticks in Europe. The prevalence of *B. valaisiana* varies geographically in questing ticks, but is generally highest in areas characterized by low vegetation and cooler climates, such as coastal Scandinavia, Scotland and alpine regions, with birds, especially migratory and woodland species, serving as its main reservoirs [1, 3–11]. *B. valaisiana* has also been detected in ticks feeding on humans in many European countries, such as Sweden [5], the Åland Islands [12], Germany [11], Slovakia [13] and Italy [14].

The pathogenic potential of *B. valaisiana* in humans is a subject of debate [2]. Most publications on this topic consist of case reports, which often provide incomplete descriptions of the clinical presentation, and the diagnosis frequently relies on methods not accepted in the European reference diagnostics of Borrelia infections [15]. For example, Rijpkema et al. 1997 identified *B. valaisiana*-like DNA (VS116 group) in erythema migrans skin biopsies from two patients [16]. Similarly, Schaarschmidt et al. 2001 detected *B. valaisiana* DNA in urine samples from six patients with unspecified symptoms [17]. One case report describes the presence of *B. valaisiana* DNA in cerebrospinal fluid of a patient with a history of spastic paraparesis [18]. Another case report identified a *B. valaisiana*-related genospecies in the blood of a patient with erythema migrans [19]. Additionally, a DNA sequence with high similarity to *B. valaisiana* strains CKA1 and VS116 was detected in the blood of a Japanese traveller with fever, diarrhoea, and arthralgia after returning from Russia [20]. Interestingly, in a prospective study conducted in 2010–2011, the occurrence of symptoms and seroconversion among tick-bitten individuals in Romania was investigated. Of the collected ticks (*n* = 389), two tested positive for *B. valaisiana*. The individuals bitten by these ticks did not develop any new symptoms, clinical manifestations consistent with a Borrelia infection, nor did they seroconvert for Borrelia antibodies during a one year follow-up period [21].

In the Tick-Borne Diseases (TBD) STING study [22], which investigated tick-bitten participants and their detached ticks in Sweden and in the Åland Islands, Finland, between 2007 and 2015, *B. valaisiana* was detected in 1.6% of ticks collected between 2008 and 2009 [23]. This study aimed to assess whether participants exposed to *B. valaisiana*-infected ticks developed Borrelia-specific antibodies, symptoms, or clinical manifestations consistent with a Borrelia infection, thereby evaluating the potential pathogenicity of *B. valaisiana* in humans.

## Materials and methods

### Study design

The TBD STING study is described in detail by Wilhelmsson et al. [23]. The study was designed as a prospective, multi-centre investigation conducted between 2007 and 2015, enrolling tick-bitten adults (>18 years) from Sweden, the Åland Islands and Norway. Participants bitten by ticks were recruited through advertisements disseminated via television, radio, newspapers, and public posters. They were encouraged to submit any ticks that had been attached to them to their local primary health‑care centre. Participants who were on antibiotic treatment during the time of inclusion, or who were receiving treatment for a disease that may influence the immune response, were excluded. Ticks detached from participants were collected, and a questionnaire encompassing tick- and health-related data [24], together with a signed informed consent, was administered at the time of enrolment. Blood samples were taken at the time of inclusion, and at the follow-up visit three months later. Additionally, all participants were instructed to seek medical care and provide further blood samples if they developed symptomatic disease during the study period. Any additional detached ticks during the study period were also collected. Finally, a second questionnaire [24] was filled out during the final visit, reporting on symptoms experienced throughout the study period and whether medical care was needed.

### Ticks and study participants

The *B. valaisiana* positive ticks included in this study were collected between the years 2008 and 2009 (*n* = 34), and had been detached from 33 participants, of whom one participant was bitten by two ticks. Participants bitten by *B. valaisiana*-positive ticks (VPOS, *n* = 33) were compared to an age- and sex matched group of participants bitten by ticks negative for *B. valaisiana* and other *Borrelia* species (VNEG, *n* = 67). Geographical areas in Sweden are described as Southernmost Sweden, South central Sweden, and Northern Sweden in analogy with a previous publication [22].

### Molecular analysis of ticks

Each tick was photographed and identified by species, developmental stage, and sex. Feeding duration was estimated for nymphs and adult females [23]. Ticks collected during the study period (at inclusion=index tick; and additional ticks collected during the study period=additional ticks) were homogenized and lysed, extracted for total nucleic acid and transcribed into complementary DNA (cDNA) as described by Wilhelmsson et al. [23]. All index ticks underwent real-time PCR analysis for *B. burgdorferi* s.l. according to Wilhelmsson et al. [5], while the additional ticks were analysed by real-time PCR according to Gyllemark et al. [25] (Table 1). Positive samples were subjected to species determination by sequencing, as previously described [23]. Index ticks were further analysed for *Anaplasma phagocytophilum*, *Francisella* spp., *Babesia* spp., Tick-borne encephalitis virus (TBEV), *Rickettsia* spp., and *Neoehrlichia mikurensis* using real-time PCR (Table 1). Additional ticks, i.e. those submitted by participants during the study period, were analysed for the presence of the same pathogens, except for *Francisella* spp. (Table 1).

**Table 1:** Primers and probes used for detection of *Anaplasma phagocytophilum*, *Babesia* spp., *Borrelia burgdorferi* sensu lato, *Francisella* spp., *Neoehrlichia mikurensis*, *Rickettsia* spp., and Tick-borne encephalitis virus in ticks by real-time PCR

Agents	Sequence (5´→ 3´)	Function	Target gene	Length (bp)	References
*Anaplasma phagocytophilum*	TTTTGGGCGCTGAATACGAT	Forward	*gltA*	64	Henningsson et al. 2015 [26]
	TCTCGAGGGAATGATCTAATAACGT	Reverse			
	**VIC**-TGCCTGAACAAGTTATG-MGBNFQ	Probe			
*Babesia* spp.	GTCTTGTAATTGGAATGATGG	Forward	*18 S* rRNA	411–452^#^	Casati et al. 2006 [27]
	TAGTTTATGGTTAGGACTACG	Reverse			
*Borrelia burgdorferi* sensu lato	GAC TCG TCA AGA CTG ACG CTA AGT	Forward	*16 S* rRNA	131	Wilhelmsson et al. 2010 [5]
	GCA CAC TTA ACA CGT TAG CTT CGG TAC TAA C	Reverse			
	GCT GAG TCA CGA AAG CGT AG	Forward	16 S rRNA	116	Gyllemark et al. 2021 [25]
	CACTTAACACGTTAGCTTCGGTA	Reverse			
	6-FAM-CGCTGTAAACGATGCACACTTGGT-MGB	Probe			
*Francisella* spp.	AAC TGG CTG ACC TTC AGC AT	Forward	*sucC*	125	Cronhjort et al. 2019 [28]
	GTG GTC GTG GTA AAG CTG GT	Reverse			
	**6-FAM** -CCG ATT AGG CTT TCT GCT ACT TCA CGA-BHQ1	Probe			
	GGG CGA ATC ACA GAT TGA ATC	Forward			
	GCG GTT CCA AAC GTA CCA A	Reverse			
	**Texas Read** -TTT TTA TGT GTC CGC CAC CAT CTG GAT C-BHQ1	Probe			
*Neoehrlichia mikurensis*	GTAAAGGGCATGTAGGCGGTTTAA	Forward	*16 S* rRNA	107	Labbé Sandelin et al. 2015 [29]
	TCCACTATCCTCTCTCGATCTCTAGTTTAA	Reverse			
*Rickettsia* spp.	TCGCAAATGTTCACGGTACTTT	Forward	*gltA*	74	Stenos et al. 2005 [30]
	TCG TGCATT TCT TTCCATTGTG	Reverse			
	6-FAMTGCAATAGCAAGAACCGTAGGCTGGAT G-MGBNFQ	Probe			
Tick-borne encephalitis virus	GGG CGG TTC TTG TTC TCC	Forward	*16 S* rRNA	68	Schwaiger and Cassinotti et al. 2003 [31]
	ACA CAT CAC CTC CTT GTC AGA CT	Reverse			
	**6-FAM** -TGA GCC ACC ATC ACC CAG ACA CA-BHQ1	Probe			
	GGC TTG TGA GGC AAA AAA GAA	Forward	*E gene*	87	Gaumann et al. 2010 [32]
	TCC CGT GTG TGG TTC GAC TT	Reverse			
	HEX-AAG CCA CAG GAC ATG TGT ACG ACG CC-BHQ1	Probe			

Bp; base pair; spp.; species

### Serological analyses

Serum samples from both inclusion and follow-up visits were screened for Borrelia-specific antibodies using two commercial enzyme-linked immunosorbent assay (ELISA) kits; C6 Lyme ELISA™ (Immunetics Inc., Cambridge, MA) and IDEIA Borrelia IgG (Oxoid, Cambridgeshire, UK) [33]. Seroconversion, i.e., the development of new Borrelia antibodies, was defined as either a change from seronegative in the inclusion sample to seropositive in the follow-up sample or as a minimum of 1.8-fold rise in Lyme index (C6 assay) or arbitrary units (IDEA assay) between the inclusion and follow-up sample [34]. All cases of suspected seroconversion or significant increase of antibody levels as indicated by the C6 assay were confirmed by using the RecomLine Borrelia IgG immunoblot (Mikrogen, Neuwied, Germany) [22, 33]. The diagnosis of Borrelia infection was predefined prior to study inclusion and was established either through documented seroconversion or by identifying a clinically confirmed Borrelia infection in medical records for participants who sought healthcare during the study period.

### Statistical analysis

SPSS Statistics (IBM SPSS version 29) was used for statistical analyses. For comparison of continuous data between VPOS and VNEG, the Mann-Whitney U test was used. Chi-2-test and Fisher’s exact test were used for comparison of categorical variables. Spearman’s rho was used for correlation analyses (seroconversions and symptoms). A p-value < 0.05 was considered significant.

## Results

### Study participants

The median age in both the VPOS (*n* = 33) and VNEG (*n* = 67) groups was 67 years. The proportion of women in the VPOS group was 58% and 60% in the VNEG group. Participants in the VPOS and VNEG groups were similarly distributed geographically: 12 (36%) and 26 (38%) from the Åland Islands, 12 (36%) and 24 (36%) from South-central Sweden, 8 (24%) and 15 (22%) from Southernmost Sweden, and 1 (3%) and 2 (3%) from Northern Sweden, respectively.

### Self-reported symptoms

Data in questionnaires on newly developed symptoms were available for 29 of 33 participants in the VPOS group. Six of 29 (21%) reported symptoms during the three-month study period, but none of them sought medical care (Table 2). None of the participants in the VPOS group reported newly developed skin lesions or were diagnosed with *erythema migrans*. Among participants in the VNEG group, 14 out of 67 (21%) reported symptoms during the three-month study period. Of these, five (7.5%) indicated in the questionnaire that they had sought medical care; however, medical records were identified for only two (3%) individuals, neither of whom received a diagnosis of tick-borne infection. None of the individuals in the VPOS group, bitten by co-infected ticks (additional pathogens, besides *B. valaisiana*), reported symptoms or sought medical care during the study period. Conversely, one (1.5%) participant in the VNEG group, who had been bitten by an index tick infected with *Rickettsia* spp., reported symptoms and sought medical attention, ultimately receiving a diagnosis of Bell’s palsy. The most common symptoms in the VPOS group were numbness (13.8%) and myalgia/arthralgia (10.3%), followed by headache (6.9%) and neck pain (6.9%) (Table 2). In the VNEG group, the most common symptom was myalgia/arthralgia (17.9%), followed by numbness (13.4%), fatigue (13.4%), headache (11.9%), and radiating pain (10.4%). No statistically significant differences in the presence of symptoms were found between the two groups (*p* = 1.0).

**Table 2:** Summary of the study participants bitten by a *Borrelia valaisiana*-positive tick (VPOS) and who reported symptoms within the three-month study period

Region of study participant	Sex	Age at inclusion	Self-reported symptoms	Sought medical care	Seroconversion
South central Sweden	F	62	Numbness	No	No
South central Sweden	F	46	Headache	No	No
Southernmost Sweden	F	67	Headache, fatigue, neck pain, cognitive difficulties, myalgia/arthralgia	No	No
Southernmost Sweden	M	64	Myalgia/arthralgia, numbness	No	Yes*
South central Sweden	F	56	Neck pain, nausea, vertigo, radiating pain, myalgia/arthralgia, numbness	No	No
Åland Islands	M	56	Numbness	No	No

F= female, M= male. * Bitten by a *Borrelia* spp.-positive additional tick

### Ticks

#### Index ticks

Of the 34 VPOS‑positive ticks identified, all were *I. ricinus*. Among them, 18 (53%) were nymphs, 15 (44%) were adult females, and one (3%) was an adult male. No larval ticks tested positive for *B. valaisiana*. One VPOS tick was co-infected with *N. mikurensis*, one with *Babesia* spp. and three with *Rickettsia* spp., respectively. No other *Borrelia* spp., besides *B. valaisiana*, were detected in the VPOS ticks. Among the VNEG ticks—those negative for all *Borrelia* spp.—*Babesia* spp. was detected in two specimens and *Rickettsia* spp. in one specimen. Comparison of tick blood-feeding time (median in the VPOS group was 32.5 h [IQR = 29] and 35 h [IQR = 43] in the VNEG group) showed no statistically significant difference between the two groups (*p* = 0.19).

#### Additional ticks

**Fig. 1:**
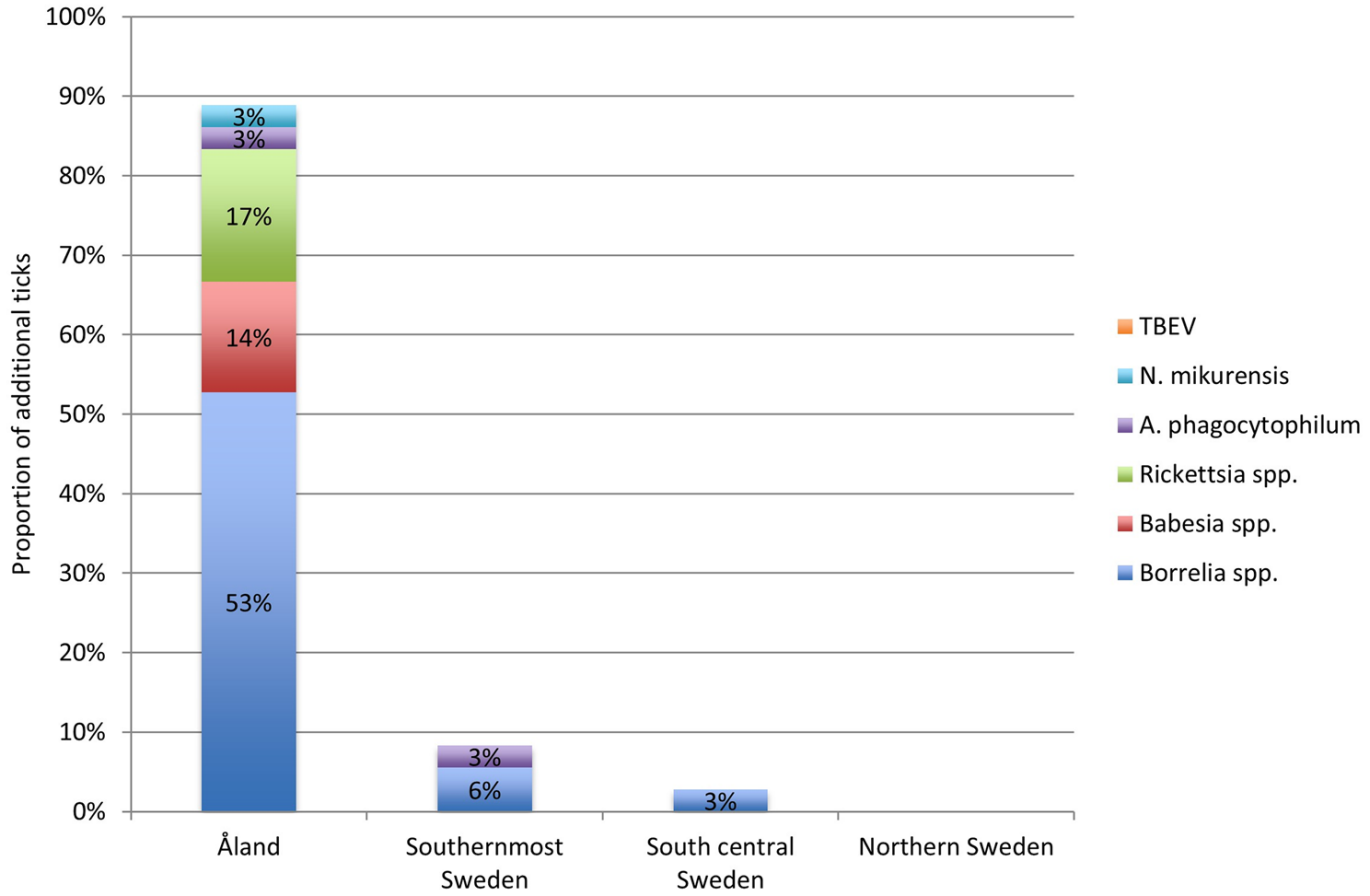
Proportion of additional ticks (*n* = 36) in the VPOS group with pathogen content, distributed by region and by the detected pathogen. TBEV; Tick-borne encephalitis virus, N; *Neoehrlichia*, A; *Anaplasma*, spp.; species

Over the three‑month study period, participants submitted an additional 190 *I. ricinus* ticks, 127 of which were provided from VPOS participants. In total, 8.4% of the ticks were classified as larvae, 82% as nymphs, 4.2% as adult females, and 1% as adult males. For 4.7% of the specimens, the developmental stage could not be determined because they were damaged during removal. Most of the additional ticks (*n* = 143) were collected by residents in the Åland Islands. In the VPOS group, 36 (28%) of the additional ticks were infected by pathogens distributed as follows: *Borrelia* spp. 22 (17.3%), of which the sequenced species included one (0.8%) *B. valaisiana*, three (2.4%) *B. afzelii* and one (0.8%) *B. garinii*; two (1.6%) *A. phagocytophilum*, six (4.7%) *Rickettsia* spp., one (0.8%) *N. mikurensis* and five (3.9%) *Babesia* spp. No ticks were infected with TBEV (Fig. 1). The additional tick positive for *B. valaisiana* did not contain any other *Borrelia* spp.

### Serological analyses

Serological data were available for 31 out of 33 (94%) participants in the VPOS group, and for 66 out of 67 (99%) participants in the VNEG group. In the VPOS group, six of 31 (19%) fulfilled the criteria for seroconversion compared to three of 66 (4.6%) in the VNEG group, with a statistically significant difference between the groups (*p* = 0.02). In the VPOS group, two (33%) of the participants who seroconverted were bitten by *Borrelia* spp.-positive additional ticks. If these two individuals are excluded from the comparison of seroconversion between VPOS and VNEG, statistical significance is no longer achieved (*p* = 0.20). The participant who was exposed to an additional tick infected with *B. valaisiana* showed no evidence of seroconversion and did not develop any clinical symptoms. In the VNEG group, one seroconverted participant was exposed to B. afzelii in an additional tick. Self-reported symptoms (any, if present) did not significantly correlate with seroconversion (*p* = 0.52). A summary of participants (and including ticks) in the VPOS and VNEG groups, who reported symptoms and/or showed seroconversion are provided as supplementary information (Table S1).

## Discussion

This exploratory study aimed to investigate whether exposure to ticks infected with *B. valaisiana* is associated with the development of symptoms, infection and/or disease in tick-bitten participants, in comparison to exposure to ticks negative for *Borrelia* spp. While no significant differences in symptom occurrence were observed between the two tick-exposed groups, individuals bitten by *B. valaisiana*-positive index ticks exhibited a significantly higher rate of seroconversion, indicative of infection, compared to those bitten by index ticks negative for *B. valaisiana*. It is noteworthy, though, that none of the *B. valaisiana*-exposed participants sought medical care for symptoms that occurred during the study period, which were mainly influenza-like symptoms and apparently resolved spontaneously. It is unknown what happened to the study participants after the study period, whether they developed symptoms afterwards and/or sought medical care for symptoms related to the tick bite. Moreover, since the study excluded immunosuppressed individuals, it remains unclear whether exposure to *B. valaisiana* may give rise to other or more severe symptoms in patients with compromised immune status. Our results align with previous findings, demonstrating that, while a small number of individuals bitten by Borrelia-positive ticks develop Borrelia-specific antibodies, an even smaller number go on to develop a symptomatic Borrelia infection [22, 33]. Non‑specific symptoms, such as headache, myalgia, arthralgia, and fatigue, are frequently observed among individuals who have been bitten by ticks [35] and are also reported by middle-aged and older adults [36, 37]. Given this overlap, and in the absence of information regarding the duration of symptoms, we cannot rule out the possibility that the symptoms reported by the participants were attributable to factors unrelated to tick bites. Given that the median age among participants in this study was 66 years, this may have had an impact on the reported symptoms. Considering that there was no difference in the presence of symptoms between the VPOS and VNEG groups, it is difficult to determine which potential symptoms may have been caused by *B. valaisiana* among the seroconverted VPOS participants.

It is important to interpret seroconversion data with caution in individuals who are frequently exposed to ticks, particularly in highly endemic regions such as the Åland Islands, where participants encountered numerous additional ticks during the study period. Furthermore, participants may have been bitten by a tick infected with *Borrelia* spp. either prior to study inclusion or during the study period without noticing it, as it is well established that fewer than 50% of patients with disseminated Borrelia infection recall a preceding tick bite [38]. In the VPOS group, six participants were exposed to additional ticks carrying *Borrelia* spp., which could not be sequenced due to high cycle threshold (ct) values indicating low pathogen load. Two of these participants displayed seroconversion, and when they were excluded from the statistical comparison of seroconversion between the VPOS and VNEG groups, a significant difference was no longer evident. The possibility that co-occurring *Borrelia* spp. other than *B. valaisiana*, present at lower bacterial loads, contributed to infection cannot be excluded and should be considered when interpreting the seroconversion findings. One participant was exposed to an additional tick positive for *B. valaisiana*, without showing evidence of seroconversion. The large proportion of additional ticks collected in the Åland Islands may be due to geographic differences, with a higher risk of contracting tick bites in the Åland Islands than in Sweden, as indicated in previous studies [22]. The public´s willingness to participate in studies and local conditions may also be relevant for the results.

In the study by Wilhelmsson et al. (2016) [22], a positive correlation was observed between tick feeding time and seroconversion, with a median attachment time of 46 h among participants who developed Borrelia antibodies. In this study, the median tick feeding time among VPOS participants was slightly shorter (32.5 h), which may perhaps have influenced the rate of seroconversion in participants exposed to *B. valaisiana*.

Co-infections involving *N. mikurensis*, *Rickettsia* spp., and *Babesia* spp. were detected among the *B. valaisiana*-positive ticks collected at inclusion, whereas no co-pathogens were identified in the additional *B. valaisiana*-positive tick. It is important to note, however, that a substantial proportion (17/22) of the additional ticks in which *Borrelia* spp. were detected could not be sequenced due to high ct values, thereby limiting species-level identification. Consequently, the possibility that some of these *Borrelia* spp. include *B. valaisiana* cannot be ruled out. Interestingly, studies on field-collected *I. ricinus* nymphs from Denmark [10] and Germany [39] indicate that *B. valaisiana* is most frequently co-detected with *B. garinii*. Moreover, co-infection with *B. spielmanii* and *B. valaisiana* has also been reported in German tick populations [40].

The prospective design of this study, including systematic blood sampling, structured medical data collection, carefully selected and relevant control subjects, analysis of additional ticks, and participant follow-up, constitutes a major strength. However, the limited number of participants bitten by *B. valaisiana*-positive ticks represents a constraint in terms of statistical power and generalisability. Although participants showed seroconversion, the three-month follow-up period may have been insufficient to capture symptoms correlated to late-onset manifestations of possible *B. valaisiana* infection, such as arthritis or *acrodermatitis chronica atrophicans*, which typically develop over a longer time frame. Studies with longer follow‑up periods are therefore warranted.

This study did not demonstrate that *B. valaisiana* causes severe disease in immunocompetent adults. Nonetheless, the higher rate of seroconversion observed among VPOS participants suggests that infection may occur, indicating a potential for pathogenicity. These findings add new knowledge regarding the human relevance of *B. valaisiana*. However, the extent to which *B. valaisiana* poses a clinical risk remains uncertain and warrants studies employing alternative designs—for example, investigations of transmission of *B. valaisiana* into tick‑bitten skin, and sequencing of Borrelia DNA‑positive clinical samples. Furthermore, comparing seroconversion rates following exposure to *B. afzelii*‑, *B. garinii*‑, and *B. valaisiana*‑positive ticks would provide valuable context for assessing the pathogenic potential of *B. valaisiana*.

## Supplementary Information

Below is the link to the electronic supplementary material.

Supplementary Material 1

## Data Availability

The datasets used and/or analysed during the current study are available from the corresponding author on reasonable request.
