# Supplementary material for: Borrelia valaisiana – a candidate human pathogen? Insights from the tick-borne diseases STING study
Source: BMC Infect Dis. 2026 May 22;26:1015. doi: 10.1186/s12879-026-13620-z (PMC13200422; doi:10.1186/s12879-026-13620-z)
Supplement: Supplementary file 1 — Supplementary Material 1 [file 12879_2026_13620_MOESM1_ESM.docx]

**Borrelia valaisiana – A Candidate Human Pathogen? Insights from the Tick-Borne Diseases STING study**

Julia Levin, Malin Lager, Marika Nordberg, Dag Nyman, Pia Forsberg, Per-Eric Lindgren, Anna J Henningsson, Peter Wilhelmsson, Johanna Sjöwall

**Corresponding author**: Johanna Sjöwall; Department of Infectious Diseases, Vrinnevi Hospital, Norrköping, Sweden; Department of Biomedical and Clinical Sciences, Linköping University, Linköping, Sweden.

[johanna.sjowall@liu.se](mailto:johanna.sjowall@liu.se)

**Table S1**. Characteristics of study participants bitten by *Borrelia valaisiana*-positive ticks (VPOS) and Borrelia-negative ticks (VNEG) who reported symptoms and/or exhibited seroconversion during the study period, along with the corresponding pathogen content of inclusion and additional ticks collected throughout the study.

| **VPOS** |  |  |  |  |  |  |  |
| --- | --- | --- | --- | --- | --- | --- | --- |
| **Region** | **Sex (M/F)** | **Age (year at inclusion)** | **Symptoms** | **Seroconversion (Yes/No)** | **Inclusion tick: pathogen content other than B.val.** | **Number of additional ticks** | **Additional ticks: pathogen content** |
| South central Sweden | F | 69 | No | Yes | No | 0 | NA |
| South central Sweden | M | 68 | No | Yes | No | 4 | No |
| South central Sweden | F | 62 | Numbness | No | No | 0 | NA |
| South central Sweden | F | 46 | Headache | No | No | 0 | NA |
| South central Sweden | F | 56 | Neck pain, nausea, vertigo, radiating pain, myalgia/arthralgia, numbness | No | No | 0 | NA |
| Northern Sweden | M | 54 | No | Yes | No | 0 | NA |
| Southernmost Sweden | F | 67 | Headache, fatigue, neck pain, cognitive difficulties, myalgia/arthralgia | No | No | 1 | No |
| Southernmost Sweden | M | 64 | Myalgia/arthralgia, numbness | Yes | No | 5 | Borrelia spp. (n=1) |
|  |  |  |  |  |  | 0 | NA |
| Åland Islands | F | 57 | No | Yes | No | 0 | NA |
| Åland Islands | M | 56 | Numbness | No | No | 0 | NA |
| Åland Islands | M | 81 | No | Yes | No | 7 | *Borrelia* spp. (n=5), *Rickettsia* spp. (n=1), *Babesia* spp. (n=3) |
| **VNEG** |  |  |  |  |  |  |  |
| **Region** | **Sex (M/F)** | **Age (year at inclusion)** | **Symptoms** | **Seroconversion (Yes/No)** | **Inclusion tick: pathogen content** | **Number of additional ticks** | **Additional ticks: pathogen content** |
| South central Sweden | M | 79 | Headache, neck pain, myalgia/arthralgia | No | No | 0 | NA |
| South central Sweden | F | 64 | Fatigue, myalgia/arthralgia, numbness | No | No | 0 | NA |
| South central Sweden | F | 69 | No | Yes | No | 0 | NA |
| South central Sweden | M | 73 | Headache, fatigue, neck pain, vertigo, myalgia/arthralgia, numbness | No | No | 0 | NA |
| South central Sweden | F | 59 | Headache, fatigue, neck pain, cognitive difficulties, radiating pain, myalgia/arthralgia, numbness | No | No | 4 | No |
| Southernmost Sweden | F | 64 | Fatigue, radiating pain, myalgia/arthralgia, numbness | No | No | 0 | NA |
| Southernmost Sweden | M | 60 | Headache, vertigo, radiating pain, myalgia/arthralgia, numbness | No | *Rickettsia* spp. (n=1) | 0 | NA |
| Southernmost Sweden | M | 67 | Headache, fatigue, neck pain, weight loss, radiating pain, myalgia/arthralgia | No | No | 0 | NA |
| Åland Islands | F | 45 | Fever | No | No | 0 | NA |
| Åland Islands | M | 59 | Headache, fatigue, neck pain, radiating pain, myalgia/arthralgia | No | No | 2 | No |
| Åland Islands | M | 55 | Headache, myalgia/arthralgia, numbness | No | No | 0 | NA |
| Åland Islands | F | 56 | No | Yes | No | 0 | NA |
| Åland Islands | F | 72 | Fatigue, myalgia/arthralgia | No | No | 0 | NA |
| Åland Islands | M | 45 | Fatigue, radiating pain, numbness | No | No | 3 | *Borrelia* spp. (n=1) |
| Åland Islands | M | 51 | Headache, fatigue, myalgia/arthralgia, numbness | No | No | 0 | NA |
| Åland Islands | M | 73 | No | Yes | No | 11 | *B. afzelii* (n=1), *Babesia* spp. (n=1) |
| Åland Islands | F | 75 | Cognitive difficulties, radiating pain, myalgia/arthralgia, numbness | No | No | 0 | NA |

NA; not analysed, M; male, F; female, B.val.; *Borrelia valaisiana*, spp.; species, n; number.
